# Association of laryngeal and nasopharyngeal tuberculosis: a case report

**DOI:** 10.1186/1752-1947-9-2

**Published:** 2015-01-06

**Authors:** Youssef Darouassi, Mehdi Chihani, Abderrahim Elktaibi, Mohamed Mliha Touati, Karim Nadour, Amine Benjelloun, Brahim Bouaity, Haddou Ammar

**Affiliations:** 3 ENT Department, Avicenne Military Hospital, Marrakech, Morocco; 4 Department of Pathology, Avicenne Military Hospital, Marrakech, Morocco; 5 Department of Pneumology, Avicenne Military Hospital, Marrakech, Morocco

**Keywords:** Larynx, Nasopharynx, Tuberculosis

## Abstract

**Introduction:**

To the best of our knowledge, the association of nasopharyngeal and laryngeal tuberculosis has never been described before in the literature. We report here a first observation.

**Case presentation:**

We report the case of a 38-year-old Arab man who presented with an isolated hoarseness. Radiological and endoscopic examinations showed a thickening of the left lateral wall of his nasopharynx and the left vocal cord. Pathology revealed the diagnosis of tuberculosis of both localizations. He received a 6-month antituberculous chemotherapy with a satisfying uneventful evolution.

**Conclusions:**

Tuberculosis should be considered in the differential diagnosis of soft tissue masses of the head and neck, particularly when the imaging findings and clinical presentation are atypical. The diagnosis of tuberculosis is mainly based on histopathological and/or bacteriological examination.

## Introduction

Tuberculosis (TB) is an infectious disease caused by *Mycobacterium tuberculosis*. The main site involved is commonly the lung. However, there is a marked increase in head and neck infections especially in developing countries [1, 2]. Laryngeal TB (LTB), which used to be associated with advanced pulmonary infection in the last century, is now increasingly a primary site [1]. Nasopharyngeal TB (NPTB) is rare, even in endemic areas [3].

Clinical, radiological and endoscopic features of head and neck TB are not specific; they are often confused with neoplastic lesions or even incidentally discovered [1]. Positive diagnosis is based on pathology and/or bacteriology. Antituberculous chemotherapy is the main treatment of this disease [1].

To the best of our knowledge, the association of NPTB and LTB has never been described in the literature. We report here a first observation.

## Case presentation

**Figure 1:**
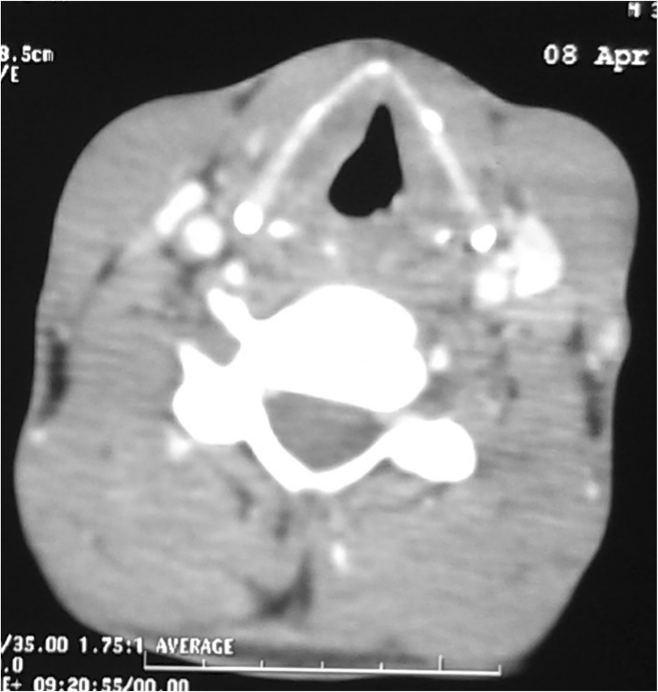
**Axial computed tomography section showing thickening of the left vocal cord.**

A 38-year-old Arab man, a heavy cigarette smoker (20 cigarettes daily for 20 years, so 20 pack years), who never worked in the wood or leather industries, with no history of diabetes, allergic rhinitis or immunodepression and with no medical history of familial or personal TB, presented with an isolated hoarseness that appeared a month prior without dyspnea or dysphagia. He did not present any nasal symptom such as obstruction or epistaxis. Moreover, he reported a non-estimated weight loss without fever or night sweats. A nasofibroscopy showed a slight thickening of the posterior wall of his nasopharynx with adenoid appearance, a budding process of the anterior commissure of the glottis and a granulomatous thickening of the left vocal cord whose mobility was reduced. His nasal cavity, oropharynx and laryngopharynx were intact. Examination showed no lymphadenopathy.A cervical and thoracic computed tomography scan showed a budding thickening of the left lateral wall of his nasopharynx and a thickening of the left vocal cord (Figure 1), with fibrosis and emphysematous bullae in both lung fields, probably linked to the heavy cigarette smoking.

**Figure 2:**
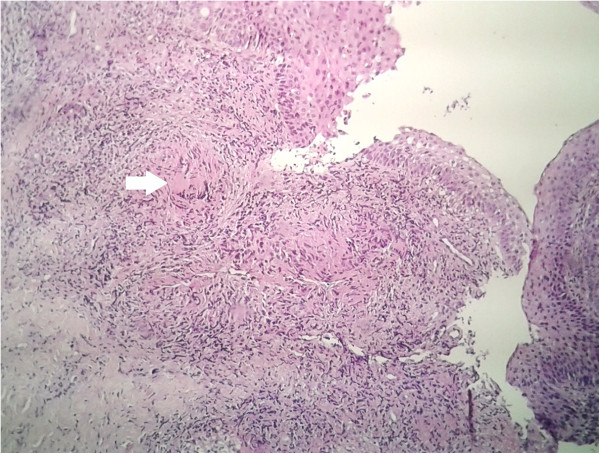
**Microscopic appearance of the laryngeal mucosa showing focal areas of epithelioid with Langhans giant cells with punctiform necrosis (arrow).** Hematoxylin and eosin ×250.

**Figure 3:**
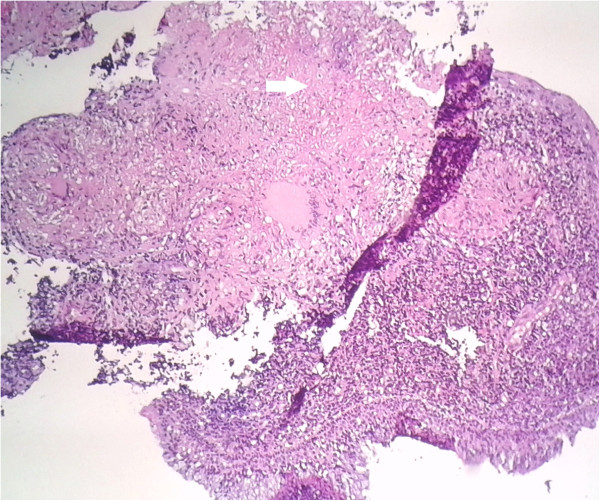
**Photomicrograph of nasopharyngeal mucosa showing epithelioid with Langhans giant cells and central caseous necrosis (arrow).** Hematoxylin and eosin staining ×250.

He underwent laryngoscopy; three biopsies of the posterior wall of his nasopharynx, a biopsy of the anterior commissure and two biopsies of the left vocal cord were performed.Histology showed on a vocal cord and nasopharynx specimen, an epithelioid and gigantocellular granulomatous process with caseous necrosis without any malignancy sign (Figures 2 and 3).

Human immunodeficiency virus (HIV) serology was negative. His complete cell blood count was normal. He was given a 6-month antituberculous treatment 2RHZE/4RH where R is rifampicin 10mg/kg/day, H is isoniazid 5mg/kg/day, Z is pyrazinamide 30mg/kg/day, and E is ethambutol 20mg/kg/day, with a satisfying uneventful evolution.

## Discussion

TB is widespread in the world with an incidence of 8.7 million in 2011 and a mortality of 1.4 million. Virtually all the organs can be affected. There has been a significant decrease in the disease in the last decades thanks to the advent of the antituberculous regimen. However, we notice a resurgence of extrapulmonary involvement partly due to HIV infection. The prevalence of HIV infection in patients with tuberculosis is about 8.8%. Up to 10% of extrapulmonary TB involves the head and neck region [4, 5] with 95% of cervical lymphadenitis. Other sites such as the larynx, pharynx, tonsils, nasal cavities, ears, sinuses, mastoids, and salivary glands can be affected but each represents less than 1% of all cases of TB [6].

Currently, LTB is rare, but prior to antibiotics, it was associated with 37% of pulmonary TB and a high mortality rate [7, 8]. In frequency, the larynx is the second site involved in the head and neck region [6] with no sex predilection. According to recent series, 50% of LTB are associated with active pulmonary infection [6]. NPTB is also rare and represents less than 1% of upper respiratory tract involvement [4]. The infection can be primary or secondary to pulmonary or systemic TB [2]. NPTB seems to be more frequent in women with two peaks of frequency, between 15 and 30, and between 50- and 60-years old [3].

When exposed to respiratory epithelium, the bacteria are phagocytized by alveolar macrophages, which are unable to digest them, subsequently allowing the unchecked multiplication of the bacteria and manifestation of disease by direct infection/extension, as well as hematogenous and lymphatic spread [8].

The larynx is rarely the primary site involved, it is rather usually infected via expectoration of sputum from tracheobronchial tree, hematogenous spread from other sites or less frequently via lymphatic spread [7, 9]. Moreover, TB of the larynx is highly contagious [10]. In the NPTB, the roof of the nasopharynx is usually the main home of the bacillus. The other walls are secondarily involved [2]. The mode of contamination does not differ from that of the larynx. However, the rich lymphatic network of the Waldeyer ring explains lymphatic nasopharyngeal contamination [3, 4].

In the past, clinical manifestations of LTB were not specific, due to the frequent association with lung disease (fever, weight loss, night sweat, fatigue and hemoptysis) [10]. Currently, the symptoms seem more evocative of larynx involvement: hoarseness (80 to 100%), odynophagia (50 to 67%), stridor and dysphagia [1, 8]. Cough, dyspnea and hemoptysis can also be present in the absence of lung infection [7]. Our patient presented with an isolated hoarseness. The most frequent form of presentation of NPTB is high jugular adenopathy (50 to 90%), followed by nasal obstruction, snoring, rhinorrhea, serous otitis, hearing loss, tinnitus, and otalgia [5] .The general symptoms can be absent [3]. Atypical presentations such as diplopia and sleep apnea have also been reported. NPTB is often underdiagnosed due to less obvious signs [4]. Our patient did not complain of nasal symptoms.

LTB is classified into four types according to its macroscopic appearance: granulomatous (like in our case), polypoid, ulcerative and nonspecific. Lesions can be unique or multiple. Granulomatous lesions are more common in patients with pulmonary TB [6]. Endoscopy in NPTB can present a varied range, from an apparently normal mucosa, to an evident mass, or a mucosa with an adenoid (like in our case) or swollen appearance, ulcers, leukoplakic areas, and various combinations [1, 3–5].

Infection by HIV should be ruled out in all patients with TB, especially if the TB is extrapulmonary and there is no apparent immunodeficiency [6].

In LTB, the four predominant patterns described based on imaging appearances include superficial ulcerative lesions, nonspecific mucosal enhancement due to inflammatory change, polypoid masses, and ulcerative/fungating lesions [11]. In NPTB, two radiological patterns have been described: a) polypoid masses, and b) diffuse thickening of the mucosa [2, 3, 5, 6]. The presence of necrosis and striped pattern in nasopharyngeal lesions, and a lack of invasion of regional structures and central necrosis with the characteristic peripheral rim enhancement of cervical lymphadenopathy are evocative of the diagnosis [2].

Mycobacterial smear and culture are difficult in these locations due to low concentrations of the bacilli. Histopathology seems to be more useful to make an early diagnosis. A biopsy must be performed in any space-occupying lesion. Sample analysis includes histology and mycobacterial culture. Histology shows granulomas with giant cells, with or without caseous necrosis. Cultures come afterward to confirm diagnosis. Quick and accurate diagnosis can also be made by polymerase chain reaction from oral samples with a high sensitivity rate (89 to 100%) [3].

TB may mimic or coexist with other conditions in the head and neck, especially tumors; therefore, tissue confirmation is mandatory [11]. It is often difficult to differentiate LTB from laryngeal cancer based on physical examination alone [7]. The danger of misdiagnosing LTB as a malignant laryngeal tumor is evident [11, 12]. The differential diagnosis of LTB includes neoplasm, syphilis, sarcoidosis, Wegener’s disease, leprosy, actinomycosis, lupus, recurrent polychondritis, rheumatoid arthritis, amyloidosis, fungal infection, chronic nonspecific laryngitis and lethal midline granuloma [6, 11]. NPTB shares many clinical features with nasopharyngeal carcinoma, including cervical lymphadenopathy [2]. Thus, making a correct differential diagnosis between these two diseases is important. Furthermore, association of cancer and TB has already been reported [2]. NPTB has also many differential diagnoses, which include malignancy (squamous cell carcinoma, lymphoma), fungal infection (aspergillosis and mucormycosis), granulomatous inflammation (sarcoidosis, leprosy and syphilis) and autoimmune disease (polyarteritis nodosa, Churg–Strauss syndrome and Wegener’s granulomatosis) [1–5].

Medical treatment consists of the combination of powerful antituberculous drugs according to the regimen: 2RHZE/4RH where R is rifampicin 10mg/kg/day, H is isoniazid 5mg/kg/day, Z is pyrazinamide 30mg/kg/day, and E is ethambutol 20mg/kg/day. A 6-month treatment is usually sufficient and results in complete recovery [7, 13]. Surgical intervention may be required in case of obstruction, abscess formation, massive bleeding, localized perforation with fistula, the failure of antituberculous medication, and/or difficulties in diagnosis [13].

## Conclusions

Head and neck TB although uncommon in developed countries, must be kept in mind in case of cervical swelling or infectious condition resistant to classical antibiotics. Given the frequency of carcinomas in this region, biopsies must be performed as much as necessary to make the right diagnosis [14].

TB should be considered in the differential diagnosis of soft tissue masses of the head and neck, particularly when the imaging findings and clinical presentation are atypical. The diagnosis of TB is mainly based on histopathological and/or bacteriological examination [1, 12, 13].

## Consent

Written informed consent was obtained from the patient for publication of this case report and accompanying images. A copy of the written consent is available for review by the Editor-in-Chief of this journal.

## Abbreviations

HIV: Human immunodeficiency virus
LTB: Laryngeal tuberculosis
NPTB: Nasopharyngeal tuberculosis
TB: Tuberculosis.
